# High autistic trait individuals do not modulate gaze behaviour in response to social presence but look away more when actively engaged in an interaction

**DOI:** 10.1002/aur.1666

**Published:** 2016-07-19

**Authors:** Elisabeth A. H. von dem Hagen, Naomi Bright

**Affiliations:** ^1^ School of Psychology, Cardiff UniversityCardiffCF24 4QHUK; ^2^ MRC Cognition & Brain Sciences UnitCambridgeCB2 7EFUK

**Keywords:** autism spectrum, social interaction, eye gaze, faces, theory‐of‐mind

## Abstract

Autism is characterised by difficulties in social functioning, notably in interactions with other people. Yet, most studies addressing social difficulties have used static images or, at best, videos of social stimuli, with no scope for real interaction. Here, we study one crucial aspect of social interactions—gaze behaviour—in an interactive setting. First, typical individuals were shown videos of an experimenter and, by means of a deception procedure, were either led to believe that the experimenter was present via a live video‐feed or was pre‐recorded. Participants' eye movements revealed that when passively viewing an experimenter they believed to be “live,” they looked less at that person than when they believed the experimenter video was pre‐recorded. Interestingly, this reduction in viewing behaviour in response to the believed “live” presence of the experimenter was absent in individuals high in autistic traits, suggesting a relative insensitivity to social presence alone. When participants were asked to actively engage in a real‐time interaction with the experimenter, however, high autistic trait individuals looked significantly less at the experimenter relative to low autistic trait individuals. The results reinforce findings of atypical gaze behaviour in individuals high in autistic traits, but suggest that active engagement in a social interaction may be important in eliciting reduced looking. We propose that difficulties with the spatio‐temporal dynamics associated with real social interactions rather than underlying difficulties processing the social stimulus itself may drive these effects. The results underline the importance of developing ecologically valid methods to investigate social cognition. ***Autism Res*** *2017, 10: 359–368*. © 2016 The Authors Autism Research published by Wiley Periodicals, Inc. on behalf of International Society for Autism Research.

## Introduction

Difficulties in social interactions are a core characteristic of Autism Spectrum Disorders (ASD). Since eye gaze plays a critical role in regulating social interaction and communication, it is not surprising that abnormalities in gaze behaviour during social interactions form part of standardised diagnostic criteria for ASD such as DSM‐5 [APA, 2013]. Interestingly, however, research into gaze behaviour in ASD, in particular patterns of looking at the eyes and mouth, has revealed conflicting results, with some research suggesting that individuals with ASD avoid the eyes and have difficulty using gaze cues appropriately whereas other research suggests typical gaze behaviour in ASD [for reviews, see Falck‐Ytter & von Hofsten, 2011; Nation & Penny, 2008]. One potential reason for discrepancies amongst previous research studying the cognitive underpinnings of gaze behaviour in ASD or social interactions more generally is that experimental paradigms have used different types of stimuli, for instance static images of faces or videos of social scenes. Furthermore, few studies have had any scope for real social interaction [Falck‐Ytter, Carlstrom, & Johansson, 2015; Hanley et al., 2015; Nadig, Lee, Singh, Bosshart, & Ozonoff, 2010; Noris, Nadel, Barker, Hadjikhani, & Billard, 2012]. Using well‐controlled stimuli, like images or videos, clearly has its benefits by allowing for precise experimental manipulation. However, they are necessarily limited in the extent to which the findings generalise to naturalistic settings. In recent years, there has been growing awareness of the need for a more ecologically valid approach to the study of social perception [Kingstone, 2009; Teufel et al., 2012]. In particular, the interactive aspects of social settings have been highlighted as a crucial but much neglected variable in social neuroscience [Schilbach et al., 2013].

As an illustration of this point, a recent study found that participants' gaze behaviour is qualitatively different when placed in a room with a live confederate compared to a videotape of the same confederate, suggesting that viewing static images or videos of social stimuli like faces does not provide an accurate reflection of social information processing in a real social setting [Laidlaw, Foulsham, Kuhn, & Kingstone, 2011]. In particular, the authors found that participants looked less at the confederate who was in the same room with them compared to the videotaped confederate. They concluded that what determines social information sampling is not the characteristics of the stimulus but the potential for direct social interaction.

These results might have important implications for our understanding of social information processing in patient populations and particularly in autism [Schilbach, 2016]. In the current experiments we, therefore, adopted an individual‐differences approach and studied gaze behaviour in a group of typical participants in realistic social interaction settings where we manipulated the potential for interaction or believed social presence, as well as whether the interaction required active participation on the part of the observer. We were specifically interested in whether the extent of autistic traits in participants modulated their looking behaviour in these different situations. We first examined the effect of believed social presence and potential for interaction on gaze behaviour by presenting participants with two videos, one of which they believed was pre‐recorded and one of which they believed was a live video‐feed of an experimenter in another room—in fact, both were pre‐recorded. In this experiment, participants were passive recipients of social input from the video; they were asked to observe and listen to the experimenter in the video, but they did not have to be an active social agent themselves. We then examined gaze behaviour during a semi‐structured active social interaction with the experimenter via a live video‐feed. Since there is some evidence to suggest that direct and averted gaze are processed differently at the cortical level in individuals with ASD [Pitskel et al., 2011; von dem Hagen, Stoyanova, Rowe, Baron‐Cohen, & Calder, 2014], as well as engaging attention differently in typical individuals with a high number of autistic traits [Chen & Yoon, 2011], we also varied the experimenter's direction of gaze systematically, throughout the video recordings as well as the live interaction, in order to provide participants with both direct and averted gaze.

Based on previous research [Laidlaw et al., 2011], we predicted that individuals would spend less time looking at the experimenter during passive viewing of a video they believed to be live relative to pre‐recorded, and that this effect would be greater when experimenter gaze was direct relative to averted. In addition, we predicted that these effects would be more striking in high autistic trait individuals. Similarly, we anticipated that during a real social interaction requiring active engagement, participants high in autistic traits would be much less likely to look at the experimenter than low autistic trait individuals.

## Methods

### Participants

54 typical participants took part in two experiments. Participants were paid and provided informed consent. They were recruited from the MRC Cognition & Brain Sciences Unit (CBU) Volunteer Panel. None of the participants had an ASD diagnosis. All participants were asked to complete the Autism Spectrum Quotient (AQ) questionnaire [Baron‐Cohen, Wheelwright, Skinner, Martin, & Clubley, 2001] after completion of the study. For the AQ analyses, the top 1/3 scorers (“high AQ group,” AQ > =20) and the bottom 1/3 scorers (“low AQ group,” AQ = <13) were used. The AQ range of these groups was determined using the data from experiment 1 and, for consistency, the same range was used in experiment 2. Participants also completed the Spielberger State‐Trait Anxiety Inventory (STAI) [Spielberger, 1983], and the Social Phobia Inventory (SPIN) [Connor et al., 2000]. Participants' scores from these questionnaires were significantly correlated with AQ scores (all *P* < 0.05).

For experiment 1, 13 participants were excluded: 9 participants' eyetracking was extremely poor or failed in one or both videos (due to glasses, double corneal reflex, poor calibration, etc.), 3 participants did not believe the deception, and 1 participant did not complete the AQ. The remaining 41 participants (23 males) had an average age of 25 ± 7 years (mean ± SD). For the AQ analyses, there were 13 participants in the low AQ group (AQ range 2–13, 5 males) and 13 participants in the high AQ group (range 20–35, 8 males).

For experiment 2, 9 participants were excluded: 8 participants' eyetracking was extremely poor and 1 participant did not complete the AQ. The remaining 45 participants (23 males) had an average age of 25 ± 6 years. For the AQ analyses, there were 14 participants in the low AQ group (5 males) and 16 participants in the high AQ group (8 males).

### Experimental Design

**Figure 1:**
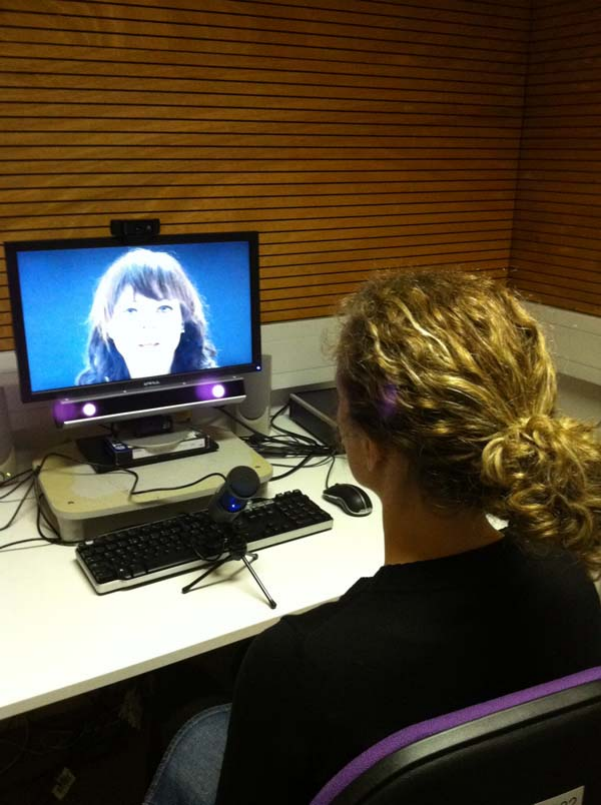
Image of the experimental setup. Participants sat in front of a widescreen monitor, which could display either pre‐recorded videos or a live video‐feed of an experimenter in a separate room. A webcam was installed above the monitor and a desktop eyetracker sat below the monitor. A microphone was placed in front of the participant. The experimenter sat in front of an identical setup in a separate room, but without an eyetracker.

A testing session involved participation in two experiments. Participants were told they would be interacting via a live video‐feed with an experimenter in another room. They were shown the other room and introduced to the experimenter who would be interacting with them. The experimenter sat in front of a widescreen monitor (1280 × 720 pixels) with a USB microphone (Audio‐Technica AT2020), and a webcam (Logitech HD 1080p) fixed to the top. In a separate room, participants had an identical setup, but their eye movements were also monitored using a 50 Hz desktop eyetracker (RED, SensoMotoric Instruments SMI) which sat below the monitor (Fig. 1).

#### Experiment 1

The purpose of experiment 1 was to study whether participants' beliefs regarding the social presence of another person might influence their passive viewing behaviour independently of stimulus characteristics. We showed participants pre‐recorded videos of an experimenter telling a short story, giving us full experimental control over stimulus characteristics, but manipulated their belief as to whether they were viewing the experimenter “live” via the video‐feed or as a pre‐recorded video. Participants were told they would hear two stories/videos and they should listen carefully as they would later be asked what they could recall; one story would be recounted live by the experimenter in the other room via the video‐feed, and the other story was a pre‐recorded video recounted by the experimenter who was sitting in the room with the participant. As described above, in fact both stories were pre‐recorded. Therefore, the only difference between these two conditions was they were *believed* to be live or not. We took several measures to ensure that the deception was as compelling as possible. Specifically, prior to viewing the videos, participants were told the video‐feed had to be “tested” and they were briefly connected live to the experimenter in the other room to say hello. This brief interaction between the participant and the person they would later see on the pre‐recorded video was important in establishing the belief that one of the videos was “live.” Moreover, immediately prior to the “live” video, the experimenter who was with the participant left the room to “warn” the other experimenter that they were ready to start the “live” video‐feed. Finally, care was taken to ensure that the experimenter's appearance and the experimental room were as shown in the video.

The order of videos, the stories, and which of the two videos was “live” (and, therefore, also which experimenter was in the room with the participant and which was in the other room) were all counterbalanced.

There were four pre‐recorded videos, average length 52.5 sec. Each video was a recording of one of two female experimenters telling one of two short stories. The first story was based on the logical memory section of the Wechsler Memory Scale [WMS‐IV, Wechsler, 2009], and the second story was designed to be similar in style and approximately matched for emotional content. The stories were matched for length (126 words). Throughout the video, the experimenter spent roughly the same amount of time maintaining direct gaze with the participant (i.e., looking directly into the webcam) and averting their gaze by looking to the left or to the right of the webcam. The experimenters' gaze was always direct or averted at the same points in time for each story. Two videos (one of each story by different experimenters) were presented to each participant using E‐prime 2.0 presentation software. Prior to each video, a calibration of the eyetracker was run, followed by an instruction screen, which reminded participants whether the upcoming video was “live” or “pre‐recorded”

#### Experiment 2

Experiment 2 used the same physical setup as experiment 1, but participants were actively engaged in a live interaction with the experimenter in the other room via the live video‐feed (this was always the same experimenter from the video they believed was “live” in experiment 1). The video‐feed was run using streaming software (Wowza Media Systems) on the MRC CBU's internal network. Prior to beginning the interaction, the eyetracker was calibrated again. The interaction was a semi‐structured, 3‐min conversation, during which the experimenter asked questions about work/study, hobbies, etc., but adjusted follow‐up questions according to what the participant said. As in experiment 1, the experimenter maintained direct gaze (looking directly into the webcam) approximately half the time and averted their gaze to the left or to the right for half the time. The timing of direct and averted gaze blocks was dictated by a Matlab script that ran on a laptop in the room with the experimenter. This program randomly sampled direct or averted gaze blocks of 5, 10, or 15 sec in length and, unbeknown to the participant, informed the experimenter about where to look by changing the screen's colour. Allocated gaze blocks were chosen such that the total length of the interaction was always 3 min and the total amount of direct and averted gaze was always 90 sec each. For averted gaze blocks, the experimenter looked at the top left or right corner of the monitor. All interactions started with a block of direct gaze.

Following experiment 2, participants' recall of the two stories from experiment 1 was assessed.

At the end of the study, participants were asked if they had noticed anything strange during the study. The aim was to determine whether the experimenter's eye movements seemed unnatural to the participant and whether they believed the deception in experiment 1. Participants were then asked explicitly about the eye movements and were told about the deception. Only three out of 54 participants reported that they had not believed the deception, and none of the participants reported anything odd about the eye movements. Participants were fully debriefed.

### Data Analysis

Eyetracking data were analysed using SMI software (BeGaze Version 3.3.56), which uses a dispersion based algorithm for detecting fixations. The minimum fixation duration was 80 ms and maximum dispersion value 100 pixels. The amount of eyetracking data for each participant relative to the length of each video or interaction was determined as the sum of the duration of all fixations and saccades over the total duration of the video/interaction. Participants, for whom the eyetracker failed to pick up a signal for more than half the time of the video/interaction, were excluded from analyses. Regions‐of‐interest (ROIs) were created around the experimenter's eye and mouth region. Most previous studies that investigated eye gaze during realistic social interactions in ASD and the typical population focussed on gaze behaviour relating to the face as a whole [Falck‐Ytter et al., 2015; Freeth, Foulsham, & Kingstone, 2013; Nadig et al., 2010; Noris et al., 2012]. We were interested in looking more specifically at potential differences in how individuals look at certain parts within the face itself. Given the debate over whether individuals with ASD show atypical gaze behaviour not only with respect to the eyes but also the mouth [Falck‐Ytter & von Hofsten, 2011; Hanley et al., 2015; Norbury et al., 2009], we decided to use the eye and mouth regions. Data were normally distributed and parametric tests were used throughout.

#### Experiment 1

The videos were blocked into experimenter direct and averted gaze blocks. Net dwell time (NDT) as a % of total trial duration in each ROI was determined for each gaze block (direct, averted) and each video condition (“pre‐recorded,” “live”). NDT includes the sum of durations of all fixations and saccades that hit the ROI, thus incorporating total time spent within the ROI. A repeated‐measures analysis of variance (ANOVA) was conducted with ROI (eyes, mouth), video condition (“pre‐recorded,” “live”), and experimenter gaze direction (direct, averted) as within‐subjects factors. For the AQ analysis, an ANOVA was conducted with ROI, video condition, and experimenter gaze direction as within‐subjects factors and AQ group (low, high) as a between‐subjects factor.

In order to ensure that any effects we observed in experiment 1 were not due to the stories themselves, participants' recall of the stories' content was quantified by two experimenters by counting the number of “story units” recalled, where each story unit encompasses a critical descriptor essential to retelling the story, for example, noun, verb, or adjective [breakdown into story units similar to Wechsler, 2009]. The average of both experimenters' scores for each participant was used to perform a paired *t*‐test on story recall. An ANOVA was also conducted to look at potential differences between the low and high AQ groups, with story as within‐subjects factor and AQ group as a between‐subjects factor.

#### Experiment 2

The video of the interaction was blocked manually for each participant into experimenter direct and averted gaze blocks. NDT as a % of total trial duration in each ROI was determined for each gaze block (direct, averted). An ANOVA was conducted with ROI (eyes, mouth) and experimenter gaze direction (direct, averted) as within‐subjects factors. For the AQ analysis, an ANOVA was conducted with ROI and experimenter gaze direction as within‐subjects factors and AQ group (low, high) as a between‐subjects factor. In all ANOVAs, Bonferroni‐corrected post‐hoc *t*‐tests were run to determine the source of significant interactions.

In order to characterise the interaction of experiment 2 in more detail, the video was also blocked into listening, thinking and speaking blocks, from the participants' perspective. Here, “thinking” is the period of time between listening (experimenter speaking) and speaking (participant speaking). NDT as a % of total trial duration in each ROI was determined for each block (listening, thinking, speaking). An ANOVA was conducted with ROI (eyes, mouth) and participant activity (listening, thinking, speaking) as within‐subject factors. For the AQ analysis, an ANOVA was conducted with ROI, participant activity, and AQ group as factors. In addition, the proportion of time spent speaking relative to the duration of the total interaction was determined and a *t*‐test performed to look at whether there were group differences between the low and high AQ groups.

## Results

### Experiment 1 – Passive Viewing of ‘Pre‐Recorded’ versus ‘Live’ Video

**Figure 2:**
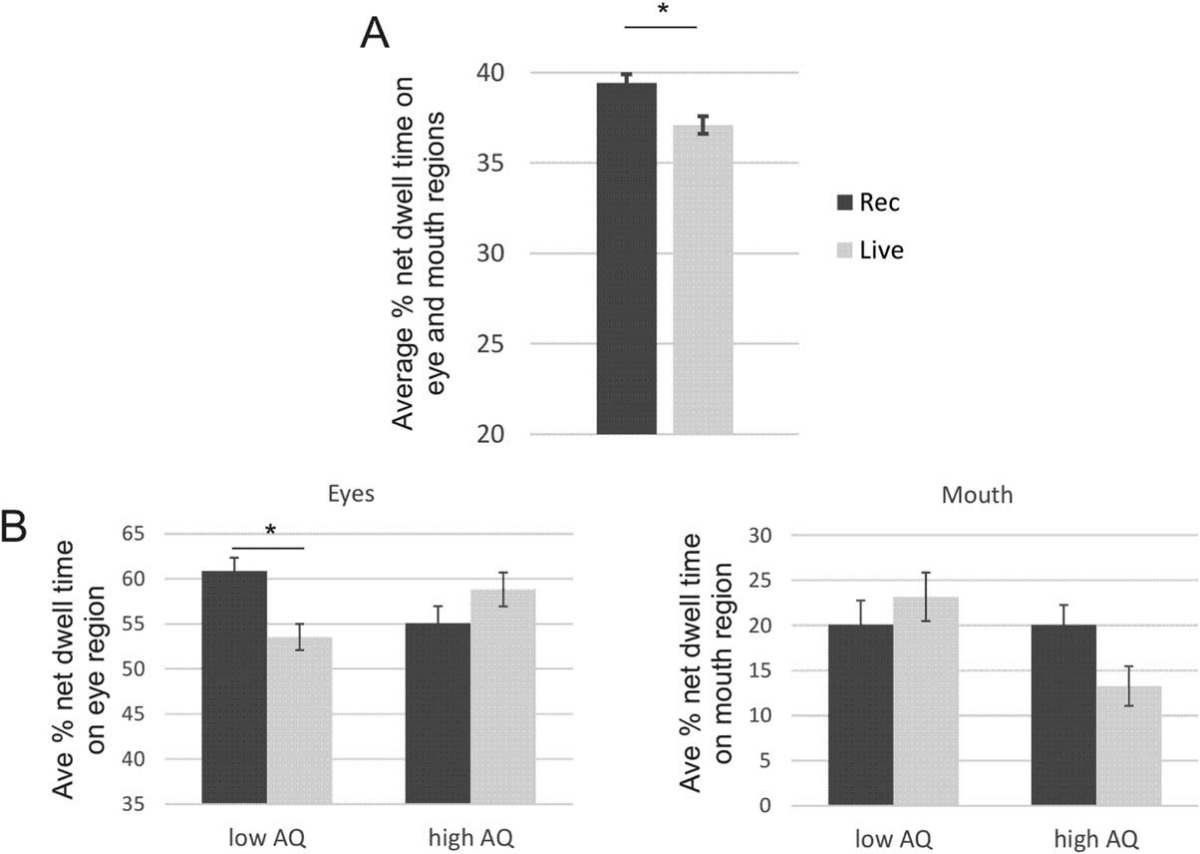
(A) Significant difference in average % net dwell time on the eye and mouth regions of the experimenter's face during the videos participants believed were “pre‐recorded” (Rec) or “live” (Live). Participants looked significantly less at the eyes and mouth when they believed the experimenter was “live.” (B) Plot of the ROI by video condition by AQ group interaction. Average % net dwell time on the eye region (top) and the mouth region (bottom) in the low and high AQ groups for videos they believed were “pre‐recorded” or “live.” Post‐hoc *t*‐tests revealed a significant difference in the amount of time spent looking at the eyes in the low AQ group for the “pre‐recorded” and “live” videos. The horizontal bars with stars denote significant differences at *P* < 0.05. All error bars depict the standard error, adjusted for within participants design according to Cousineau [2005]. Note each plot displays a slightly different range on the *y*‐axis to better illustrate the observed differences.

The purpose of experiment 1 was to examine gaze behaviour during passive viewing of two pre‐recorded videos while the believed social presence of the person in the videos was manipulated. We compared gaze behaviour when participants viewed a video they believed was pre‐recorded versus a video they believed was “live.” Regardless of video condition, overall, participants spent more time looking at the eyes (NDT mean *M* = 58.3%, standard error SE = 4.0%) than at the mouth of the experimenter (*M* = 18.2%, SE = 2.8%) (main effect of ROI, *F*(1,40) = 38.51, *P* < 0.001, partial eta squared $\eta_{p}^{2}=0.491$). More importantly and as we had predicted, there was a main effect of video condition (*F*(1,40) = 5.73, *P* = 0.021, $\eta_{p}^{2}=0.125$), such that participants spent less time looking at the experimenter in the video they believed to be live relative to the video they believed to be pre‐recorded (Fig. 2A). Finally, there was a video condition by experimenter gaze direction interaction (*F*(1,40) = 4.88, *P* = 0.033, $\eta_{p}^{2}=0.109$). Post‐hoc *t*‐tests revealed no significant differences (*P*'s > 0.2) in the amount of time participants looked at the experimenter when the experimenter's gaze was direct or averted, regardless of video condition.

When participants were split into high (AQ > =20) and low AQ (AQ = <13) groups, there was again a main effect of ROI (*F*(1,24) = 25.44, *P* < 0.001, $\eta_{p}^{2}=0.515$), such that, regardless of AQ group, overall participants spent more time looking at the eyes than the mouth. Interestingly, however, there was a borderline three‐way interaction between ROI, video condition, and AQ group (*F*(1,24) = 3.68, *P* = 0.067, $\eta_{p}^{2}=0.133$). Post‐hoc *t*‐tests revealed that the interaction was driven by the low AQ group spending significantly less time looking at the eyes when they believed the video was live compared to when they believed it was pre‐recorded (*P* = 0.039) (Fig. 2B). This was not the case for the high AQ group, who showed no significant difference in time spent looking at the eyes regardless of whether they thought the video was live or pre‐recorded (*P* = 0.275). When participants' state anxiety scores were included as a covariate in the model, there was still a borderline significant ROI by video condition by AQ group interaction (*P* = 0.056). However, when trait anxiety or SPIN were included as covariates, the interaction was no longer significant (*P* > 0.18).

There was a significant difference in participants' recall of the content of story A (on average 14.77 remembered story units) relative to story B (12.94 units; *t*(40) = 3.13, *P* = 0.003, Cohen's d = 0.211). This finding cannot, however, account for any of the effects reported above given that both stories were equally often used in all conditions. There was no interaction between AQ group and story recall (*F*(1,24) = 1.460, *P* = 0.239).

### Experiment 2 – Active Engagement in Real‐Time Social Interaction

In experiment 2, we examined gaze behaviour while participants were actively engaged in a social interaction with the experimenter in real‐time. Across all participants, there was a main effect of ROI only (*F*(1,44) = 18.04, *P* < 0.001, $\eta_{p}^{2}=.291$) with participants spending more time looking at the eyes (*M* = 41.73%, SE = 3.15) than the mouth (*M* = 19.13%, SE = 2.56). There was no effect of experimenter gaze direction (*F*(1,44) = 1.473, *P* = 0.231), nor any interaction between gaze direction and ROI (*F*(1,44) = 0.135, *P* = 0.715).

**Figure 3:**
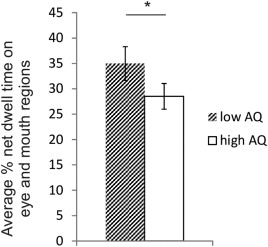
Significant difference in average % net dwell time on the eye and mouth regions of the experimenter's face for low and high AQ groups during the live interaction. Low AQ participants spent significantly more time looking at the eyes and mouth than high AQ participants. Error bars depict the standard error.

**Figure 4:**
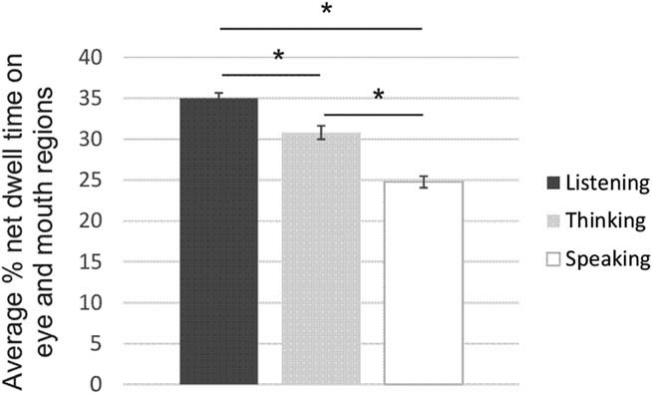
Average % net dwell time on eye and mouth regions when participants were listening, thinking or speaking during the live interaction with the experimenter. Participants spent significantly more time looking at the eye and mouth regions when they were listening than when they were thinking or speaking. Similarly, they spent significantly less time looking at the eyes and mouth when speaking than when thinking. Error bars depict the standard error, adjusted for within participant's design.

When participants were split into high and low AQ groups, there was again a main effect of ROI (*F*(1,28) = 13.78, *P* = 0.001, $\eta_{p}^{2}=0.330$) with participants looking more at the eyes (*M* = 43.78%, SE = 6.48) than the mouth (*M* = 19.71%, SE = 3.17). Importantly, there was also a main effect of AQ group (*F*(1,28) = 6.188, *P* = 0.019, $\eta_{p}^{2}=0.181$) with the low AQ group spending more time looking at the eyes and mouth than the high AQ group (Fig. 3). There was no ROI by group interaction (*F*(1,28) = 1.092, *P* = 0.305). When participants' state anxiety scores were included as a covariate in the model, there was still a main effect of AQ group (*P* = 0.05). However, when trait anxiety or SPIN were included as covariates, there was no longer a main effect of AQ group (*P* > 0.14). Since people tend to look away more when speaking than listening, and the relative time spent speaking versus listening was not controlled for across participants, we also broke down the live interaction into listening, thinking, and speaking blocks from the participants' perspective. As expected we found a significant difference in the amount of time participants spent looking at the eyes and mouth when listening, thinking and speaking (*F*(2,88) = 32.906, *P* < 0.001, $\eta_{p}^{2}=0.428$) (Fig. 4), but there was no interaction with AQ group (*F*(2,56) = 0.123, *P* = 0.885). Nevertheless, we also performed an ANOVA (ROI by experimenter gaze direction by AQ group) with proportion of time spent speaking included as a covariate. We still found a main effect of AQ group (*F*(1,27) = 5.712, *P* = 0.024, $\eta_{p}^{2}=0.175$) such that the low AQ group spent more time looking at the experimenter (*M* = 35.14%, SE = 28.37) than the high AQ group (*M* = 28.37%, SE = 1.87). All other main effects and interactions were not significant (*P*'s > 0.17).

## Discussion

In experiment 1 of the current study, we assessed the impact of another person's social presence on viewing behaviour, and how that behaviour is affected by autistic traits. Replicating previous findings [Laidlaw et al., 2011], we found that overall participants looked less at the experimenter in the video they believed to be live compared to the one they believed to be pre‐recorded, suggesting that the mere potential for social interaction affects gaze behaviour. Interestingly, however, high autistic trait individuals did not significantly change their looking behaviour according to the experimenter's (believed) social presence, neither did they show reduced looking at the experimenter overall relative to individuals with low autistic traits. The absence of an effect in the high AQ group was surprising and suggests that they were relatively insensitive to the potential for social interaction.

In experiment 2, we studied looking behaviour when participants were actively engaged in a real‐time social interaction with the experimenter. Here, high autistic trait individuals looked significantly *less* at the experimenter than low autistic trait individuals. While the literature on gaze behaviour during active social interactions is mixed, some previous studies have found similar results in ASD [Auyeung et al., 2015; Noris et al., 2012]. Our experiments could not be directly compared because they necessarily had to differ in various respects other than the manipulation of interest (passive vs. active). Nevertheless, the contrasting results in experiments 1 and 2 suggest that while the mere social presence of another person is sufficient to reduce gaze directed at this person in low autistic trait individuals, an important factor driving reduced eye gaze at another person in high autistic trait individuals may be active engagement in a social interaction. Previous research has placed emphasis on faces themselves driving atypical gaze patterns in ASD, whether due to hyperarousal [e.g., Dalton et al., 2005] or indifference/disinterest in faces [Dawson, Webb, & McPartland, 2005]. By contrast, our results suggest that atypicalities in social information processing in high autistic trait individuals are not related to the social stimulus itself (e.g., the face or other person) [Cusack, Williams, & Neri, 2015; Sevgi, Diaconescu, Tittgemeyer, & Schilbach, 2016]. Rather, these individuals seem to adjust their gaze behaviour when dealing with the complexities of a real‐time social interaction requiring their active engagement.

While the bulk of the literature on gaze behaviour in individuals with ASD used static pictures of faces or video‐recorded stimuli, some previous studies investigated gaze behaviour during direct interactions [Auyeung et al., 2015; Falck‐Ytter et al., 2015; Hanley et al., 2015; Nadig et al., 2010; Noris et al., 2012]. Importantly, however, experiment 1 of our study also explicitly focussed on the role of social presence, directly comparing conditions in which another person was (believed to be) socially present with those, in which they were not. Laidlaw et al. [2011] previously showed reduced social orienting in the typical population towards a live compared to a video confederate. We have extended this finding in several critical ways. Our “live” and “recorded” conditions differed only in participants' beliefs regarding social presence and the potential for interaction, since both were video recordings. This manipulation thus means our experiment had full experimental control. By contrast, Laidlaw et al. [2011] compared a real person in a room with a video recording, which are two physically very different stimuli. Our results suggest that, rather than necessitating the physical presence of another person, when presented with identical video stimuli, typical participants' beliefs alone about social presence are sufficient to affect their gaze behaviour.

Contrary to expectations, individuals high in autistic traits, despite also believing the deception, showed no change in their viewing behaviour. In this group, beliefs regarding social presence do not appear to affect gaze behaviour. One way of conceptualising the difference between the “recorded” and the “live” stimulus is that they are associated with different mental state inferences [Teufel et al., 2012]. In order for changes in gaze behaviour to occur, mental state inferences are made regarding the experimenter: for example, “she can see me” for the “live” stimulus, and these modulate behaviour in a top‐down manner. As difficulties with theory‐of‐mind or making mental state inferences regarding another person are thought to be core deficits in ASD [Baron‐Cohen, Leslie, & Frith, 1985], it may be that high autistic trait individuals do not make the same, or indeed any, mental state inferences regarding the experimenter in the different conditions, and as a result there is no top‐down modulation of their gaze behaviour.

While there was an apparent lack of modulation of gaze behaviour during passive observation of videos in high autistic trait individuals, active engagement in a real‐time social interaction with the experimenter significantly reduced gaze directed towards the experimenter. This finding is consistent with work by Auyeung et al. [2015], who looked at the effect of oxytocin on eye contact behaviour in adults with ASD during a video‐based interaction. In their study, the experimenter maintained direct gaze throughout, and individuals with ASD showed reduced looking at the eyes relative to controls for the placebo condition. They interpreted their results as evidence for eye‐contact (i.e., direct gaze) avoidance in ASD. Our results, however, suggest that, in the typical population with high autistic traits, looking less at the eye region is not an avoidance of eye contact per se since the effect we observed was independent of gaze direction. In addition, we observed no reduction in looking at the eyes when high autistic trait individuals did not have to actively engage with the experimenter and were only watching and listening (as in Experiment 1). Instead, it seems that the social engagement and reciprocity required in a real interaction with another person is important in eliciting the viewing behaviour typically associated with ASD [Schilbach, 2016].

One functional reason for averting gaze during an active social interaction is to reduce cognitive load—faces are rich in information, and, particularly when speaking, individuals avert their gaze more in order to cope with the increased cognitive load associated with, for example, planning speech or drawing information from memory [Doherty‐Sneddon, Bruce, Bonner, Longbotham, & Doyle, 2002; Glenberg, Schroeder, & Robertson, 1998]. One possible explanation for the differences in gaze behaviour between the high and low AQ groups during the active interaction may be that high autistic trait individuals find active engagement in a social interaction more cognitively demanding than low autistic trait individuals. Although we found no differences in the amount of time spent looking at the experimenter as a function of AQ group for the listening, speaking and thinking phases, which are associated with differing cognitive loads, we nevertheless found an overall reduction in time spent looking at the experimenter in the high AQ group. Since a key component underlying successful and fluent interaction is the synchronisation or coordination and timing of the interacting partners, it is possible that individuals with high autistic traits have greater difficulties with the spatio‐temporal dynamics of a real‐time social interaction which might impose higher cognitive demand on these individuals and thus lead to greater gaze aversion overall. While motor coordination and timing difficulties have previously been reported in ASD, further research is needed to address whether these difficulties may lie at the core of their social communication atypicalities.

A further aspect of the data not mentioned above is the absence of reduced looking at the experimenter overall in the high AQ group in Experiment 1. While a large body of research suggests that individuals with ASD show reduced gaze even in response to static or video‐recorded stimuli of faces [e.g., Klin, Jones, Schultz, Volkmar, & Cohen, 2002; Riby & Hancock, 2009a, 2009b), this finding has been contested by others [e.g., Freeth, Ropar, Chapman, & Mitchell, 2010; Kuhn, Kourkoulou, & Leekam, 2010; van der Geest, Kemner, Camfferman, Verbaten, & van Engeland, 2002]. The current experiments support the latter, adding to studies that suggest that a reduction in gaze in response to static or video‐recorded stimuli is not as robust a finding in ASD as is often believed. One possible explanation raised in a recent meta‐analysis of eye‐tracking studies of social stimuli in ASD is that reduced social attention in ASD is greatest with greater social content (more than one person/face in stimulus)[Chita‐Tegmark, 2016]. The meta‐analysis was not, however, able to draw any conclusions on the effect of real social interactions or passive versus active engagement in an interaction.

Contrary to expectations, we found little effect of experimenter eye gaze direction on participants' gaze behaviour in either experiment. This contrasts with a previous study by Freeth et al. [2013] which found that experimenter eye contact did affect participants' eye movements when engaged in a face‐to‐face interaction with the experimenter. It is difficult to directly compare these results with ours, as their periods of experimenter direct or averted gaze were static over long fixed time periods rather than changing over the course of the interaction as ours did. However, one other reason for the different results may be the physical presence of a person in front of the participants in their study, whereas our participants interacted with the experimenter via video‐feed. Eye contact may be given greater significance and have a larger effect on gaze behaviour in a face‐to‐face setting.

## Limitations

In this study, we tested a total of 54 participants, of which a large number remained after excluding those with poor eye tracking data. Some of our findings are based on this full sample. However, due to the nature of the research design, we were left with relatively small sample sizes for the analyses relating AQ to gaze behaviour. As a result, the scope and generalisability of these findings into the ASD population might be limited. In addition, the small sample sizes prevented us from investigating any potential confounds associated with participants' gender, which may also impact looking behaviour.

Another important point relates to the analyses including trait anxiety and social phobia. In our sample, autistic traits, trait anxiety, and social phobia were all highly correlated, which fits with the overlapping symptoms and co‐morbidity of conditions like autism, anxiety disorders, and social phobia. As a result, the findings here may also be interpreted from a slightly different perspective, with the observed behaviour being a manifestation of specific symptom clusters, rather than a specific diagnosis. In fact, this corresponds well with a more general move within translational research that emphasises a dimensional approach to the study of psychiatric conditions [Cuthbert & Insel, 2013]. Here, the notion is that a focus on symptom clusters is an important complementary alternative to studies based on categorical diagnosis, which might group biologically heterogeneous syndromes into one category, potentially thwarting attempts to understand the underpinnings of these conditions.

## Conclusions

We have shown that the believed social presence of another person is sufficient to affect gaze behaviour in typical individuals, such that participants look less at the eye and mouth regions of the experimenter they believe is “live.” Strikingly, individuals high in autistic traits did not avert their eyes more for the “live” video, suggesting that their beliefs about the social presence of another are not sufficient to modulate their gaze behaviour. However, when actively engaged in a real‐time social interaction, involving responding to the experimenter, there was a significant reduction in time spent looking at the experimenter. Our study suggests that patterns of gaze behaviour in high autistic trait individuals are dependent on the social situation, rather than just the social stimulus or presence of another person, and that reduced looking at another person in individuals with ASD in an interactive setting may be driven by the spatio‐temporal complexities associated with active engagement.
